# Kinetic and Mechanistic Release Studies on Hyaluronan Hydrogels for Their Potential Use as a pH-Responsive Drug Delivery Device

**DOI:** 10.3390/gels10110731

**Published:** 2024-11-12

**Authors:** Saliha Erikci, Niklas van den Bergh, Heike Boehm

**Affiliations:** 1 Department of Cellular Biophysics, Max Planck Institute for Medical Research, 69120 Heidelberg, Germany; 2 Institute for History and Ethics of Medicine, Heidelberg University, 69120 Heidelberg, Germany; 3 Department of Nuclear Medicine, Heidelberg University Hospital, 69120 Heidelberg, Germany; 4 Faculty of Engineering Sciences, Heidelberg University, 69120 Heidelberg, Germany

**Keywords:** hydrogel, hyaluronic acid, drug delivery, environmental pH, release mechanism

## Abstract

Hyaluronic acid, a biocompatible polymer, holds significant potential for drug delivery applications. Its variable degree of protonation, which entails tunable physical properties, makes it an ideal candidate for developing pH-sensitive hydrogels. Like other smart drug delivery systems, pH-responsive hydrogels can enhance medical treatment and expedite the healing process. However, the inherent complexity of hydrogels poses challenges in identifying suitable matrix systems. This study evaluates the potential of thiolated hyaluronic acid hydrogels, physically cross-linked with deacetylated disaccharide units of the polymer, for use in drug delivery. Using low-molecular-weight dextrans as model drugs, we investigated the system’s response to different pH environments in terms of swelling as well as the kinetic and mechanistic release of the encapsulated compound. The data suggest tunable release properties of the gel regarding drug size and pH value. Our results demonstrate the gel system’s potential for smart drug delivery. We anticipate that this system is a promising candidate for use in transdermal wound healing applications and strongly encourage further investigations using other sorts of (model) drugs to gain a more detailed insight into its pH-responsive transport qualities.

## 1. Introduction

Throughout the past few years, the design of adaptable drug delivery systems has increasingly become a goal of the development of novel materials. In particular, the release of active compounds as a response to environmental changes such as the pH represents a commonly approached strategy for the highly effective treatment of wounds [1]. Being an endogenous non-sulfated saccharide polymer, hyaluronic acid (HA) is known to function as a biocompatible material. Due to its polyanionic, linear structure consisting of repetitive disaccharide units, HA is able to efficiently bind water and several kinds of protein structures [2], which renders the polymer a crucial structural component of the extracellular matrix (ECM) contributing to wound healing processes [2,3]. In general, pH-dependent swelling of hydrogels relies on the pH of the surrounding medium relative to the acidic or basic moiety’s p*K*_a_ or p*K*_b_ [4] As for HA, the deprotonation of carboxylic acid moieties occurs within a medium of pH > p*K*_a_ = 3, generating fixed negative charges on the polymer chains and mobile positive charges in the solution [5]. This leads to an increase in hydrophilicity, the number of immobilized negative charges, and electrostatic repulsion between the chains [5,6], resulting in a higher uptake of water and thus swelling of the hydrogel [7].

Since drug delivery systems can differ in many ways, such as their molecular structure or behavior toward different kinds of stimuli [8], it is essential to keep in mind that varying the matrix material and environmental conditions will lead to different release kinetics. Finding a way to model the release of a drug in order to compare different parameters is therefore crucial to identify the most suitable one for the application of the desired drug. While, in general, there are no restrictions when it comes to finding the best fitting function to a set of data, some models have proven to suit drug release data more often than others (Table 1). A broadly used semi-empirical model was established by Higuchi [9]. As a special case of the Korsmeyer–Peppas model [10,11,12], it depicts the ratio of target concentration *c_t_* to the maximum target concentration *c*_∞_ in the surrounding medium after when approaching infinity as a function of the square root of the time *t*. The rate constant *k* varies from model to model.

The exponent *n* allows conclusions regarding the type of release kinetics and is dependent on the hydrogel’s geometry (Table 2). A solely diffusion-caused release is described by Fick’s law, whereas the zero-order model, also termed *Case II transport*, describes linear non-Fickian kinetics, stating that the release is controlled by relaxation or swelling of the matrix. Values in between these extremes are considered hybrid release mechanisms and are called anomalous [12,14]. Values of *n* that are lower than the tabular Fickian diffusion value still indicate a diffusion-controlled mechanism [15,16]; however, they can indicate a hampered variation of transport [17]. Other models, like Peppas–Sahlin’s [13], are able to estimate the proportion of Fickian and Case II contributions by superposition of the two. Lastly, there are several non-empirical equations, such as first-order [10,18] and second-order [18], as well as logarithmic models that can correspond to the raised data.

**Figure 1:**
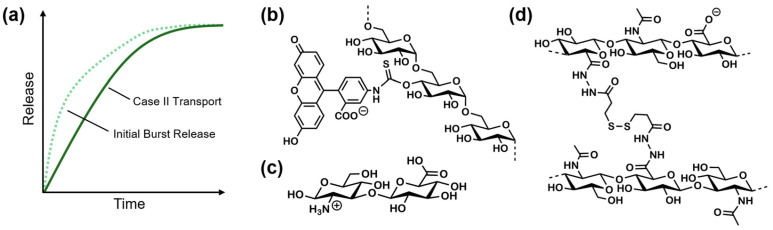
(**a**) Schematic diagram of an initial burst in a zero-order drug release profile (Case II transport, adapted from Huang and Brazel [19]). (**b**) Cutout of the chemical structure of FITC-dextran. (**c**) Chemical structure of dHA^+^ cross-linker. (**d**) Cutout of the chemical structure of the analyzed cross-linked HA hydrogel matrix according to Erikci et al. [35].

In some cases, finding an appropriate model for drug delivery devices can be particularly challenging. These circumstances can often be traced back to a phenomenon called *initial burst* [19]. This effect evokes a prompt release of large amounts of drug before approaching a more stable rate fitting one of the aforementioned equations (Figure 1a). Reasons for initial burst releases can be diverse and vary with the processing conditions; for instance, the effect may be controlled by the drug’s solubility in the release medium [20]. Furthermore, devices that were loaded via incubation in drug-containing solutions often show surface adsorption of drug molecules. However, devices such as hydrogels loaded during the gelation step also happen to display initial bursts, which can be caused by drug molecules diffusing to the surface of the device prior to adding any release medium. Although burst-release phenomena are commonly portrayed as unpleasant, they do have positive aspects. For instance, they enable useful applications in wound treatment strategies and offer ways to establish targeting delivery by introducing triggers for burst-releasing behavior [19].

Apart from the matrix composition and release mechanism, the encapsulated drug sets additional requirements for the drug delivery system. Many cross-linking strategies demand harsh gelation conditions, for instance, high-energy irradiation [21], heat [22], or the use of highly reactive [23] and oxidative compounds [24]. This might cause compatibility issues given the lack of stability of certain drugs [21]. To study release behavior, model drugs are commonly used to simulate and pre-assess interactions of similarly structured drug molecules with the matrix [25,26]. Dextrans are typically used to mimic hydrophilic molecules or particles [25]. Being α-1,6- and α-1,3-branched d-glucose polymers, they are highly water soluble and capable of forming hydrogen bonds [27]. Furthermore, they are available in a broad range of molecular weights and hydrodynamic radii [28]. When linked to fluorescein isothiocyanate (FITC, Figure 1b), dextrans are easily traceable [27]. Lastly, the structural resemblances with low- to high-molecular-weight drugs can thus help in the development of delivery systems: diuretics like mannitol [29], antidiabetics like acarbose [30], laxatives like lactose or sorbitol [31], and even anti-cancer drug candidates, such as galectin-3 inhibitors [32], are found on the lower scale of weight. Hydrophilic peptide hormones like vasopressin or insulin [33] may be mimicked by larger dextrans, and immunoglobulins like Trastuzumab and Rituximab [34] are found around 150 kDa.

In this study, we examine an HA hydrogel system (Figure 1d) previously published by Erikci et al. [35] in order to assess its possible application as a drug delivery device. The gel system relies on modified HA able to form hydrazide-based cross-linking disulfides with the assistance of physically stabilizing disaccharide cross-linker molecules (dHA^+^, Figure 1c). This way, no further additives are required to induce gelation in the presence of dextrans as model drugs.

As a consequence, this hydrogel system offers two decisive advantages: drug molecules sensitive to oxidation can now be easily embedded into the hydrogel during the gelation step, while HA provides a biocompatible and degradable polymer matrix. Compared to HA gels exhibiting different thiolation degrees, we focused on the assessment of the system’s pH responsiveness and the evaluation of its drug release mechanism. Apart from that, other features, such as swelling reversibility and drug uptake by the gel, were investigated.

## 2. Results

### 2.1. pH-Dependent Swelling

**Figure 2:**
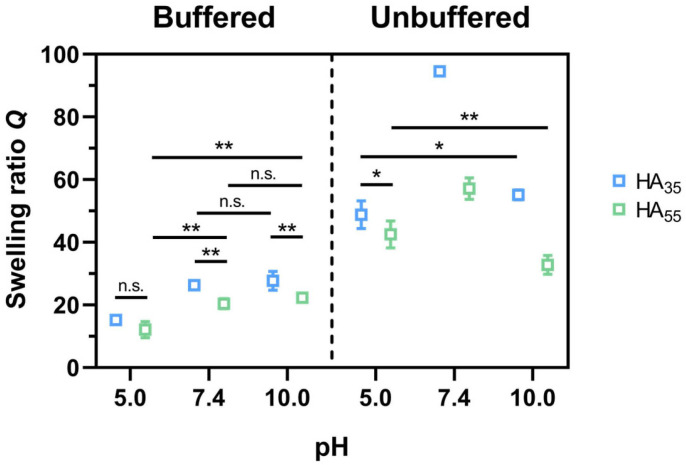
Swelling ratio *Q* (mean ± SD) of HA_35_ and HA_55_ in different unbuffered (NaOH and HCl) and buffered (150 mM citrate buffer, pH 5.0; DPBS, pH 7.4; and 150 mM borate buffer, pH 10.0) solutions (*n* = 3). For some samples, error bars are smaller than symbol size. Student’s *t*-tests were performed. For the sake of clarity, highly significant differences (*p* < 0.001) were not highlighted (*: *p* < 0.05; **: *p* < 0.01; not significant: n.s.).

**Figure 3:**
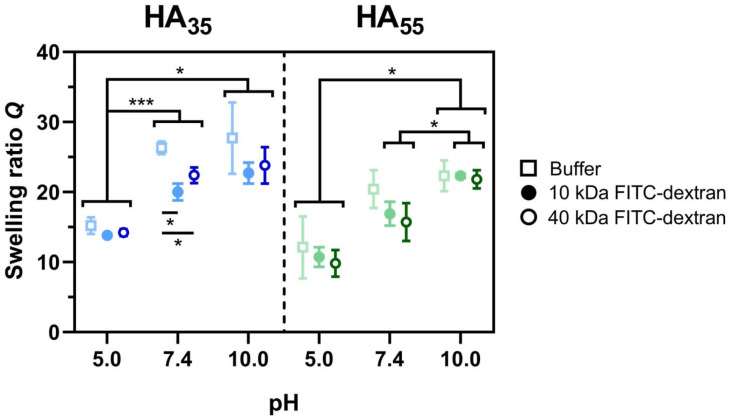
Comparison of the swelling ratios *Q* (mean ± SD) of HA_35_ (blue) and HA_55_ (green) hydrogels in different buffered solutions (150 mM citrate buffer, pH 5.0; DPBS, pH 7.4; and 150 mM borate buffer, pH 10.0) in the presence and absence (buffer only) of FITC-dextrans (*n* = 3). For some samples, error bars are smaller than symbol size. Student’s *t*-tests were performed. For the sake of clarity, only selected *p* values are shown (*: *p* < 0.05; ***: *p* < 0.001).

Due to their porosity, hydrogels are well-suited for use as drug delivery systems [36,37]. Their swelling ability can determine the diffusion of drugs through the hydrogel network while being controlled both by molecular interactions such as hydrogen bonds or electrostatic forces as well as the steric interaction between the drug and the polymer network. Since the swelling behavior of hydrogels at different pH values represents one of their major characteristics and is often connected to other material properties [35,36,37], a fundamental understanding of this phenomenon is crucial to comprehend and show correlations to other findings. Therefore, the swelling ratio for the herein-examined HA system was determined at different pH values in unbuffered and buffered solutions. These measurements included studying the influence of the degree of thiolation (Figure 2 and Figure 3) and the influence of the cross-linking agent or dye in the gel (Figure 3). According to their degree of thiolation [35], the hydrogels are designated HA_35_ and HA_55_ with respect to their cross-linking ratio of 35% and 55%. Hydrogels were incubated in each pH solution to reach constant mass, assuring an equilibrium state was achieved, and the mass of each replicate was determined (Figure 2).

Overall, hydrogels showed a higher swelling ratio when incubated in an unbuffered solution compared to buffers. As for the latter case, the swelling ratio reaches maximum values of 94.5 ± 0.7 for HA_35_ and 57.1 ± 3.4 for HA_55_ at pH 7.4. At pH 10.0, the swelling ratio decreases to 55.1 ± 1.6 for HA_35_ and 32.8 ± 3.0 for HA_55_. However, in buffered solutions, the swelling ratio constantly increases, with the pH starting at pH 5.0 from 15.2 ± 0.7 for HA_35_ and 12.1 ± 2.6 for HA_55_. Nonetheless, it appears to approach a plateau for pH 7.4 and higher, around 27 for HA_35_ and 21 for HA_55_.

**Figure A1:**
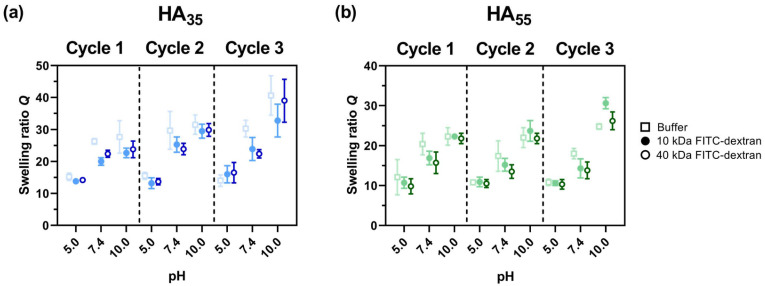
Reversibility studies for the swelling ratios *Q* (mean ± SD) of (**a**) HA_35_ and (**b**) HA_55_ hydrogels in different buffered solutions (150 mM citrate buffer, pH 5.0; DPBS, pH 7.4; and 150 mM borate buffer, pH 10.0) in the presence and absence (buffer only) of FITC-dextran (*n* = 3). For some samples, error bars are smaller than symbol size.

In addition to that, reversibility studies of three swelling cycles were performed with complete drying of the gels in between the swelling process (Figure A1). Here, the same trend was observed, with a notable increase in *Q* at pH 10.0 for the second (31.5 ± 3.0 for buffer only) and third cycle (40.6 ± 6.2 for buffer only) of HA_35_, as well as the third one of HA_55_ (24.8 ± 0.7 for buffer only). Lastly, the swelling ratios increase for lower thiolation degrees in both unbuffered and buffered solutions, with larger differences in unbuffered environments (Figure 2 and Figure A1).

As a way to determine the influence of encapsulated model drugs and dHA^+^ on medium uptake, the swelling ratios of hydrogels loaded with FITC-dextran of various molecular weights were studied (Figure 3). To exclude effects caused by a dextran concentration gradient, FITC-dextran loaded gels were incubated in dextran-containing buffered solutions.

As for Figure 2, the same trend of steadily increased swelling at higher pH values could be observed for all tested conditions. However, the presence of additional drug load inside the gel overall decreases the swelling ratio compared to buffer only, regardless of the dextran’s molecular weight. This causes the swelling ratio to reach its plateau level only at higher pH values compared to gels containing dHA^+^ only. Remarkably, at pH 7.4, HA_35_ swollen in buffered dye solution showed a significantly lower swelling ratio for either 10 kDa FITC-dextran (20.0 ± 1.2) and 40 kDa FITC-dextran (22.4 ± 1.1) compared to the buffer-only sample (26.3 ± 0.9).

### 2.2. Drug Release

**Figure 4:**
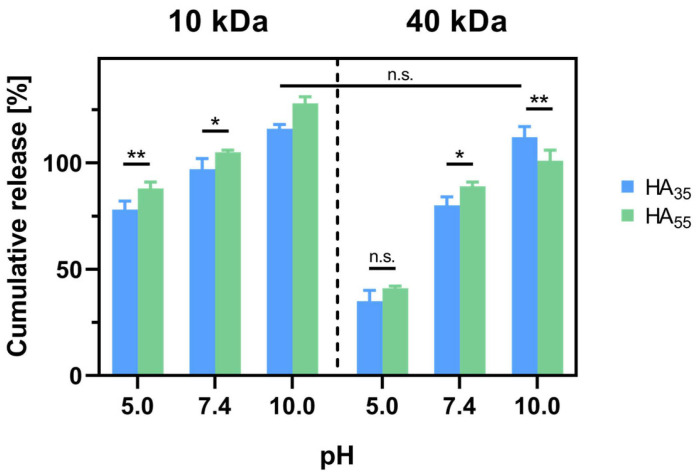
Comparison of the swelling ratios *Q* (mean ± SD) of HA_35_ and HA_55_ hydrogels in different buffered solutions (150 mM citrate buffer, pH 5.0; DPBS, pH 7.4; and 150 mM borate buffer, pH 10.0) in the presence and absence (buffer only) of FITC-dextrans (*n* = 3). Student’s *t*-tests were performed. For the sake of clarity, highly significant differences (*p* < 0.001) were not highlighted (*: *p* < 0.05; **: *p* < 0.01; not significant: n.s.).

During the process of developing materials for drug delivery, kinetic studies represent a crucial necessity to understand the system’s behavior inside the organism. For this purpose, drug release data for HA_35_ and the HA_55_ hydrogel loaded with FITC-dextrans (0.4 g/L) during gelation were gathered. The concentration of FITC-dextran in the surrounding medium at different pH conditions was monitored through fluorescence measurements. To determine the relative amount of FITC-dextran released from the different HA hydrogels, the measured FITC-dextran concentration in the surrounding medium after 24 h was considered in ratio to the calculated maximum concentration for a complete release of dye from the gel (Figure 4).

It was found that, from acidic to basic conditions, both HA_35_ and HA_55_ show a significant increase in dextran release. Furthermore, the results reveal a lower tendency in release for 40 kDa dextran, especially for pH 5.0 and still for pH 7.4. For HA_35_ at pH 5.0, a release of (78 ± 4)% was measured for the 10 kDa dextran and a release of (35 ± 5)% for the 40 kDa dextran. For HA_55_, similar numbers were observed with (88 ± 3)% and (41 ± 1)%, respectively. Regarding the degree of cross-linking of HA_35_ and HA_55_ for the individual pH values and molecular weights of the dextrans, no major differences were observed, yet HA_55_ tends to show a slightly higher release after 24 h than HA_35_. Lastly, it should be noted that for the samples measured in borate buffer (pH 10.0), exceptionally high values of apparent release were recorded, which will be discussed in more detail in the further course.

**Figure 5:**
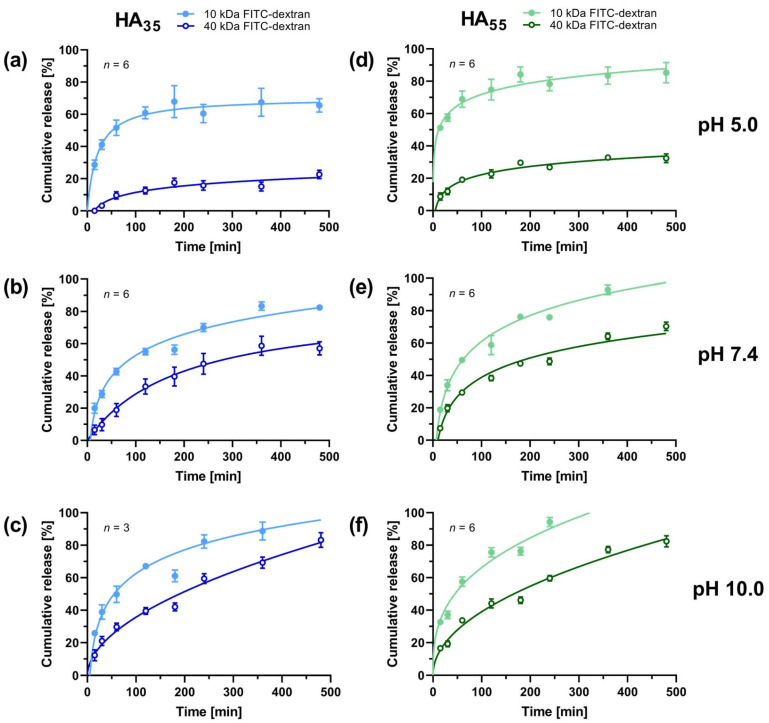
Cumulative release (mean ± SD) of FITC-dextran from HA_35_ (**a**–**c**) and HA_55_ (**d**–**f**) hydrogels at different pH values. Each graph represents the most suitable model function found by fitting the gathered data (see Table A1) to the different drug release models introduced in Table 1. For some samples, error bars are smaller than symbol size.

In order to draw conclusions about suitable kinetic release models and about the transport mechanism of the system, the release progression of FITC-dextrans was monitored via fluorescence intensity throughout the first 8 h under various pH conditions (Figure 5). Subsequently, all data sets were plotted according to the most common drug transport models (Table 1) and assessed based on their coefficient of determination (*R*^2^, Table A1).

First and foremost, it is essential to mention that for most of the data sets, more than one model emerged as appropriate to describe the release process, as *R*^2^ values did not differ drastically in some cases. All of the best-fitting models revealed an *R*^2^ value of 0.9340 (HA_35_ at pH 5.0, 10 kDa FITC-dextran) or higher (Table A1). A logarithmic model proved to be suitable for most data sets (7 out of 12), followed by Korsmeyer–Peppas (3 out of 12) and second-order transport (2 out of 12). In order to draw conclusions about the type of transport (Table 2), all data sets were fitted using Korsmeyer–Peppas’ model to determine the exponential value of *n* (Table A2). Due to the experimental setup, the geometry of the hydrogel was considered a film, as there is merely one contact surface between the gel and the buffered solution. Additionally, the values for the rate constants *k*_0_ according to Peppas–Sahlin’s model were determined (Table A2). In the further course, these findings will be discussed in more detail.

Apart from mechanistic points of view, the kinetic data complement the previously described cumulative release after 24 h (Figure 4). For all curves, more rapid release kinetics were observed for the 10 kDa dextran regardless of the pH. Nonetheless, this effect was particularly emphasized for an acidic environment at pH 5.0. Moreover, it should be highlighted that for all dextrans and degrees of thiolation tested, a plateau-like state of drastically reduced release is approached within the first 120 min.

### 2.3. Gel Loading

**Figure 6:**
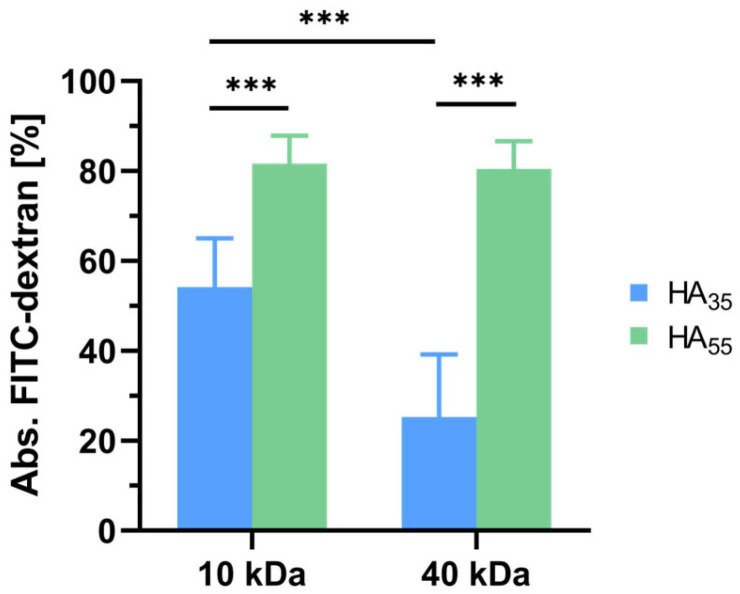
Comparison of dye loading (mean ± SD) for HA_35_ and HA_55_ hydrogels via incubation in DPBS buffered FITC-dextran solution at pH 7.4 (*n* = 9). Unpaired, two-tailed Student’s *t*-tests were performed, showing *p* < 0.001 (***) for all significant data sets.

There are two common ways of loading hydrogels: the drug can either be added during the gelation process or loaded via incubation in a drug solution [38]. To estimate the potential of the latter option for the herein-discussed system, HA_35_ and HA_55_ hydrogels were incubated in FITC-dextran solution after the gelation process. The FITC-dextran concentration of the incubation solution (0.4 g/L) was chosen to match the concentration of dextran inside the gel when loaded during the gelation step (see 2.2). After incubation, a washing step was performed to minimize the effects caused by the surface adsorption of the dextran. The amount of absorbed dye was measured through the fluorescence intensity of released FITC-dextran to the surrounding medium after 24 h (Figure 6).

The results show significant differences in loading between the two degrees of thiolation: (8.2 ± 0.6)% of loaded dextran mass for 10 kDa and (8.0 ± 0.6)% for 40 kDa FITC-dextran HA_55_ is able to take up more dye than HA_35_ with (5.4 ± 1.1)% for 10 kDa and (2.5 ± 1.4)% for 40 kDa FITC-dextran. Notably, there was no significant difference found for the two molecular weights of FITC-dextran in HA_55_; however, for HA_35_, the smaller dextran tends to achieve significantly higher loading values.

## 3. Discussion

Hyaluronic acid takes a special place amongst materials in drug delivery development. Besides its biocompatibility [2], it is known to cover a broad spectrum of physiological functions while being able to change its properties depending on its protonation state or modifications [5,35]. As the human body bears a broad range of pH environments in different tissues and fluids, pH-sensitive and other smart hydrogels represent a highly demanded category of drug delivery devices [36]. Here, we examined a physically and chemically cross-linked HA hydrogel system established by Erikci et al. [35] in regard to its adaptiveness toward different pH environments. For our experiments, we selected a broad pH range from 5.0 to 10.0 to cover most of the bodily pH environments in regard to the diverse pathogenic profiles of patient populations. A more detailed understanding of the system’s behavior, as well as physical and mechanistic properties, is supposed to help evaluate and fully exploit its potential use as a pH-responsive drug delivery device.

First of all, the hydrogels’ swelling properties were investigated both in unbuffered and buffered solutions of different pH values (Figure 2). It could be shown that in both scenarios, the hydrogel system exhibited a pH-dependent swelling behavior. For both HA_35_ and HA_55_, the least swelling was observed at a low pH (pH 5.0). While for a buffered environment, a continuous increase in swelling was reported for higher pH values, unbuffered solutions gave a maximum swelling ratio at pH 7.4 regardless of the degree of thiolation. For pH 10.0, the latter showed a decrease in its water uptake. In general, it is expected that water binding is favored in the presence of ionic hydrogel moieties, which holds true for the increased swelling at a certain pH demonstrated for HA_35_ bearing more carboxylate functions compared to HA_55_. It also explains the gradually rising amount of bound water inside the gel for a higher pH in the buffered medium since more carboxylate groups exist in their deprotonated state. However, this strictly rising trend was not seen in an unbuffered environment. One explanation for this could be a counteracting osmotic force in buffered media. Another suggested conclusion for the swelling behavior at pH 10.0 in an unbuffered solution is the emphasized leakage of the ionic cross-linker dHA^+^ in the swollen state of the hydrogel. By acidifying the solution in a feedback loop, it leads to the shrinkage of the hydrogel, resulting in less swelling at the time of measurement. The absence of this effect for buffered media further underlines the hypothesis.

Subsequently, it was examined whether the presence of FITC-dextrans acting as model drug compounds inside the gel influences the previously observed swelling properties. In fact, it could be shown that for both 10 kDa and 40 kDa dextrans, there was no significant difference in swelling for any of the three pH ranges (Figure 3). Additionally, it could be shown that for both pH 5.0 and pH 7.4, the systems’ swelling ratios were reproducible throughout at least three swelling cycles (Figure A1). At pH 10.0, an increase in water uptake was detected from the second (HA_35_) or the third (HA_55_) cycle onwards. This can be explained by the onset of partial degradation of the HA gel, which could be observed during the experimental procedure. In accordance with that, data published by Vercruysee et al. revealed the complete hydrolysis of hydrazide cross-linked HA hydrogels at pH 9.1 after four days [39]. The resulting generation of larger pores and the regeneration of carboxylate functions explains the higher tendency to take up water.

As a next step, the cumulative release after 24 h was measured in order to gain insight into the maximal amount of dextran released from the gel systems at various pH ranges (Figure 4). It was again found that this parameter is pH-dependent, showing the same trends as the swelling ratio of samples in the buffered medium. For both degrees of thiolation, an increase in cumulative release was detected with higher pH values of the buffer solution. Although this is valid for both sizes of FITC-dextrans, 40 kDa dextran showed an even more emphasized difference both for HA_35_ and HA_55_, especially comparing pH 5.0 and pH 7.4. At pH 5.0, less than 50% of 40 kDa dextran was released from either hydrogel system within the first 24 h, while practically all dextran was released at pH 10.0. This indicates stronger attractive forces between the gel and the encapsulated drug for more acidic conditions. As 40 kDa dextran offers a larger total of hydrogen bond donors and acceptors per molecule, dextran–gel interactions are more pronounced and allow for stronger captivation of the model drug inside the matrix. Moreover, the higher molecular weight contributes to reduced Brownian motion, resulting in a smaller diffusion coefficient and thus a lower diffusion rate. As mentioned before, a cumulative release of more than 100% found for samples at pH 10.0 can be again traced back to degradation of the hydrogel or FITC-dextran, as cleavage of FITC from the polysaccharide is also known to cause an increase in fluorescence intensity [40].

In the further course, the influence of the different degrees of thiolation, dextran sizes, and pH conditions on the drug transport was evaluated in a kinetic setting. All data sets were assigned the most suitable mathematical plot to model the release process (Figure 5 and Table A1). Overall, no Case II transport was observed, but the results rather suggest a (hindered) release according to Fickian diffusion. Conclusions about the type of transport (Table 2) were drawn from fitting all data sets using the Korsmeyer–Peppas model. Due to the experimental setup, the geometry of the hydrogel was considered a film, as there is merely one contact surface between the gel and the buffered solution. Since *n* ranged from 0.16 ± 0.01 (HA_55_ at pH 5.0, 10 kDa FITC-dextran) to 0.57 ± 0.06 (HA_35_ at pH 7.4, 40 kDa FITC-dextran), the data predominantly indicate a Fickian release mechanism [15,16]. For half of the data sets—mostly concerning 40 kDa dextran—a slight dependence of drug release on swelling cannot be excluded, as the values are located around the transition point from Fickian to anomalous transport. The rest of the data sets, however, indicate a (hindered) release according to Fick’s law. Similar outcomes are obtained when comparing the *k*_0_ rate constants for Peppas–Sahlin’s model [13]. Since gel loading was performed during the gelation step, initial burst effects from surface adsorption can be excluded. To estimate the significance of other burst effects, the Korsmeyer–Peppas model was fitted after adding an additional burst term to describe a possible shift upward around *t* = 0 [19]. However, no positive values were obtained for the added term, indicating the absence of initial burst effects. Nonetheless, at pH 5.0 and pH 7.4, steeper initial release slopes were recorded for more cross-linked HA_55_, which still requires an explanation. Also, for HA_55_, slightly higher release rates were found, which were most distinctive for acidic conditions. This behavior might originate from retardation effects [41] within the matrix of HA_35_ caused by higher amounts of interfering dHA^+^ chloride trying to leave the gel matrix. Thus, competitive molecules such as dHA^+^ may give rise to a longer path length for the diffusive transport of dextran. Looking at the trends seen for the swelling ratio, a rise in the pH value causes the slope of the graphs to increase as well. Here, the more significant effects observed for HA_55_ further support the retardation theory.

Lastly, the loading capacity of the gel system in dextran solution was analyzed in order to evaluate the system’s potential for a broader range of applications and functionality. It could be observed that after 24 h at pH 7.4, no HA gel system managed to approach loading capacities that are feasible by loading the drug during the gelation step. Remarkably, HA_35_ showed significantly lower loading in a buffered FITC-dextran solution than HA_55_, both for 10 kDa and 40 kDa dextrans. Nonetheless, for HA_35_, 10 kDa FITC-dextran was loaded to a significantly larger extent compared to 40 kDa dextran, a difference that was not observed for HA_55_. Since electrostatic repulsion is known to inhibit the diffusion of the drug into the matrix [38], the smaller uptake for HA_35_ can be linked to possible repulsive interactions between the carboxylate groups of hyaluronan and those of FITC in its deprotonated state. This effect seems to be less decisive for HA_55_ bearing fewer anionic groups due to the higher degree of cross-linking. On the contrary, attractive interactions like hydrogen bonds originating from the dextran’s hydroxyl functions seem to generate a driving force for the dye to be embedded into the matrix. Hydrogels with lower degrees of thiolation thus might favor surface adsorption of the dye over integration into the gel. A higher molecular weight, however, further reduces the dye’s ability to penetrate the matrix, as suggested by the results for HA_35_ and 40 kDa FITC-dextran.

## 4. Conclusions

By encapsulating a model drug represented by FITC-dextrans inside the HA matrix, we could show that the examined system has significant potential to be used as a pH-responsive drug delivery system. Its biocompatibility and its remarkable properties to withhold loaded drugs at an acidic pH and release them at neutral to basic conditions render the system an interesting delivery device candidate for various pH-controlled physiological processes. Basic pH-induced degradation of the gel structure might furthermore be advantageous in several fields of usage. In regard to this, we suggest further investigations toward applications such as a smart wound patch gel. Given the pH shift from 7.4 to 8.9 for acute and chronic wounds toward more acidic values around 4.5 to 5.3 for intact skin [42], the release qualities of the gel system appear to be within an ideal range. The simultaneous release of an acidic dHA^+^ cross-linker might even help to emphasize the retarded retention of the drug when approaching the end of the wound healing process. With hindered Fickian transport dominating the suggested kinetic release mechanism, the liberation of drug was also shown to be controlled by the molecular weight of the model drug itself, which offers further options to adapt the system’s kinetic behavior. Moreover, loading experiments underlined the benefits of cross-linker-assisted gelation, as drugs can be incorporated into the matrix during the gelation process without the worry of unwanted damage. In the tested system, this enabled higher loadings of drugs than that which could be achieved by incubation in a drug solution. In addition, this may eliminate the need to use excess amount of drugs, rendering the preparation process more efficient and less expensive.

## 5. Materials and Methods

Research-grade hyaluronic acid (average molecular weight of 74 kDa) was purchased from Lifecore Biomedical (Chaska, MN, USA). The cross-linkers for hydrogel formation were synthesized, adapted from a protocol outlined in work by Vibert et al. [43]. Hydrogels were prepared as described by Erikci et al. [35]. FITC-dextrans were obtained from Sigma-Aldrich (Darmstadt, Germany). Buffer solutions (1 L) were prepared in ddH_2_O, and their pH was adjusted using hydrochloric acid or sodium hydroxide solution and an Orion Star A211 pH meter from Thermo Scientific (Waltham, MA, USA). For the preparation of borate buffer (150 mM, pH 10.0), boric acid (9.27 g, 150 mmol) was dissolved in ddH_2_O (900 mL). For the preparation of DPBS buffer (pH 7.4), purchased DPBS buffer was adjusted to the desired pH value using sodium hydroxide solution and a pH meter. For the preparation of citrate buffer (150 mM, pH 5.0), citric acid monohydrate (12.9 g, 61.5 mmol) and sodium citrate dihydrate (26.0 g, 88.5 mmol) were dissolved in ddH_2_O (800 mL). For the preparation of Tris buffer (1 M, pH 8), Tris base (121.1 g, 1 mol) was dissolved in ddH_2_O (800 mL).

### 5.1. Gel and Cross-Linker Preparation and Determination of the Degree of Thiolation

Hydrogels and dHA^+^ cross-linker were prepared according to the previously reported procedure outlined by Erikci et al. [35]. The cross-linking rate defined by the degree of thiolation was determined using Ellman’s assay according to the aforementioned protocol. For HA_35_, a degree of thiolation of 35 ± 2% and, for HA_55_, one of 55 ± 1%, was obtained.

### 5.2. Swelling Ratio of Buffered and Unbuffered Systems

To assess the swelling ratio and its reversibility, empty Eppendorf tubes were weighed. FITC-dextran-loaded (0.4 g/L) or unloaded gels were prepared inside the Eppendorf tubes and incubated in unbuffered solution (NaOH/HCl solution at pH 5.0, 7.4, 10.0), buffer solution (150 mM citrate buffer at pH 5.0, DPBS at pH 7.4, or 150 mM borate buffer at pH 10.0), or FITC-dextran solution (0.4 g/L in 150 mM citrate buffer at pH 5.0, DPBS at pH 7.4, or 150 mM borate buffer at pH 10.0, 300 µL) for 24 h. The gels were cautiously washed with the respective solution (200 µL), and the tubes were weighed again. Buffer solution (1.8 mL) was added to the gels and removed after 24 h.

To calculate the swelling ratio *Q*, the mass of the swollen gel *m*_s_ and the mass of the dried gel *m*_d_ were obtained according to

(1) $$Q=\frac{m_{s}}{m_{d}}=\frac{m_{t,s}-m_{t}}{m_{t,d}-m_{t}}$$

by generating the difference between the tube’s mass *m*_t,s_ containing the swollen gel or the tube’s mass *m*_t,d_ containing the dried gel and the mass *m*_t_ of the empty Eppendorf tube. For reversibility studies, gels were freeze-dried in between swelling cycles.

### 5.3. Gel Loading Experiments

**Figure A2:**
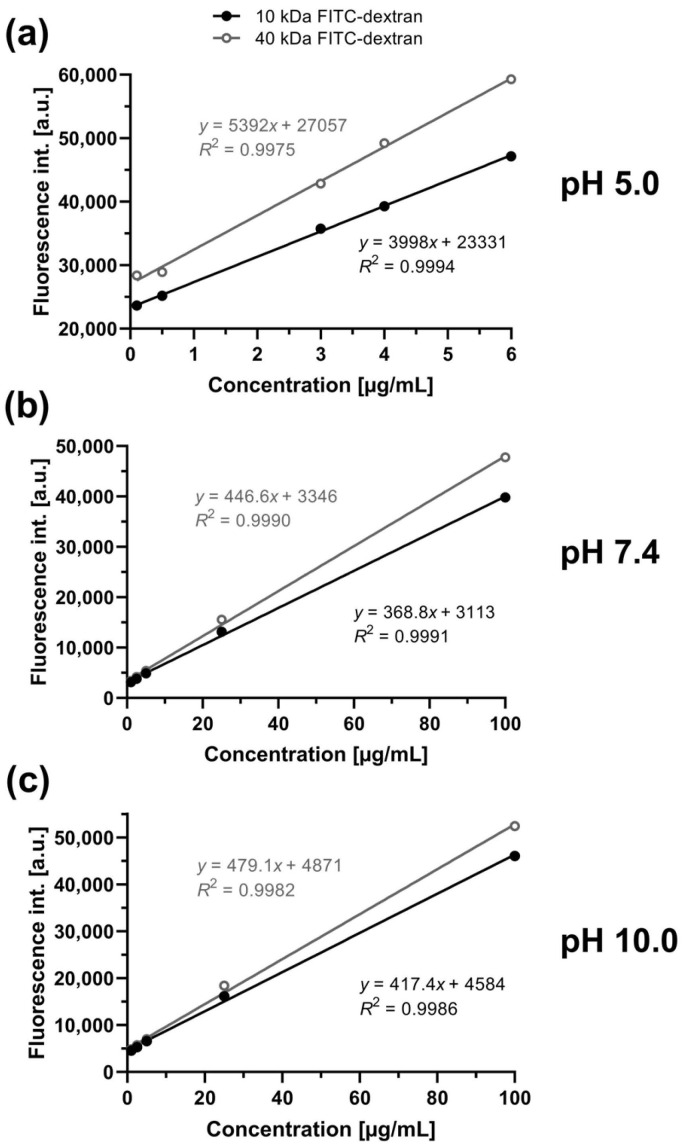
Standard curves used for the determination of FITC-dextran concentrations in buffered samples at (**a**) pH 5.0, (**b**) pH 7.4, and (**c**) pH 10.0.

To examine the ability of drug absorption, FITC-dextran solution (0.4 g/L in DPBS pH 7.4, 300 µL) was added to the hydrogel. For the control group, DPBS (pH 7.4, 300 µL) was used instead of FITC-dextran solution. After 24 h, the solution was removed, and the gel was cautiously washed with DPBS (pH 7.4, 200 µL). The gel was incubated in DPBS (pH 7.4, 1.8 mL) for another 24 h, and the fluorescence intensity was measured. To calculate the percentage of loaded dextran, 10 µg of FITC-dextran per 25 µL gel (thus corresponding to a concentration of 0.4 g/L) was set to represent 100%. To calculate the released amount of dye, standard curves (Figure A2) were used.

### 5.4. Drug Release Experiment

As a method to model continuous endogenous drug removal via the bloodstream, so-called *sink conditions* were applied in all kinetic experiments. By keeping the concentration of the drug or model drug below 10 to 20% of its solubility at every point in time, artificial saturation effects are minimized, and a chemical equilibrium is not reached within critical stages of the experiment [44]. In these studies, the solution volume was set up to be 1.8 mL to prevent the FITC-dextran from exceeding a value of more than 1.4% of its initial concentration within the gel.

For the analysis of the drug release kinetics and mechanisms, buffer solution (1.8 mL) was added to the FITC-dextran-loaded hydrogel (0.4 µg/µL or 10 µg of dextran per 25 µL gel), and fluorescence measurements were carried out after 15, 30, 60, 120, 180, 240, 360, and 480 min, as well as 24 h. For the control group, unloaded gels were used. After thorough mixing, samples (200 µL) at pH 7.4 (DPBS) and pH 10.0 (borate buffer, 150 mM) were measured directly, and the solution was transferred back to the release reservoir afterward. Samples (100 µL) at pH 5.0 (citrate buffer, 150 mM) were mixed with Tris buffer (1 M, pH 8, 100 µL) and then measured. Instead of transferring back the solution, 100 µL of citrate buffer was added to the release reservoir. To calculate the concentration of FITC-dextran at each point in time, standard curves (Figure A2) of the dextrans were established.

### 5.5. Student’s t-Test

To estimate the significance of the raised data, unpaired and two-tailed Student’s *t*-tests were performed while assuming a standard distribution of the respective data sets. The level of significance was set to *α* = 0.05.

### 5.6. Fluorescence Spectroscopy and Absorption Measurements

Absorption and fluorescence measurements were carried out using a Tecan Spark (Männedorf, Switzerland) plate reader. Unless stated otherwise, fluorescence measurements were carried out in 96-well plates (black, flat bottom) using a volume of 200 µL at an excitation wavelength of 490 nm and an emission wavelength of 520 nm. Other settings were based on the plate reader’s optimized calibrations (z-position: 19,637, Gain: 59 at pH 5.0, 45 at pH 7.4, and 47 at pH 10.0). Unless stated otherwise, absorptions at 420 nm were measured in 96-well plates (clear, flat bottom) using a volume of 300 µL. Data evaluation was performed via Microsoft Excel 16.35, OriginPro 2020b 9.7.5.184, and GraphPad Prism 8.0.1.

## Figures and Tables

**Table 1:** Common models used to describe the kinetics of drug release.

Model	Equation	Specifications
Korsmeyer–Peppas	$\frac{c_{t}}{c_{\infty }}=k_{p}t^{n}$	with *k*_h_ and *n* = 0.5: Higuchi’s model with *k*_0_ and *n* = 1: zero-order model
Peppas–Sahlin	$\frac{c_{t}}{c_{\infty }}=k_{h}t^{n}+k_{0}t^{2n}$	value of n dependent on the ratio d/l, where *d* is the diameter and *l* the thickness (height) of the gel [13]
Logarithmic	$\frac{c_{t}}{c_{\infty }}=a\;\log\left(t\right)+b$	-
First-order	$\frac{c_{t}}{c_{\infty }}=1-e^{-k_{1}t}$	-
Second-order	$\frac{c_{t}}{c_{\infty }}=1-\frac{1}{1+k_{2}t}$	*k*_2_ is dependent on the drug concentration inside the gel

**Table 2:** Exponential values *n* of drug release mechanisms for different hydrogel geometries.

Geometry	(Hindered) Fickian	Anomalous Transport	Case II Transport
Film	≤0.50	0.50 < *n* < 1.00	1.00
Cylinder	≤0.45	0.45 < *n* < 0.89	0.89
Sphere	≤0.43	0.43 < *n* < 0.85	0.85

## Data Availability

The data presented in this study are available on request from the corresponding author.

## Appendix A

To show the reversibility of the swelling process for the examined HA hydrogels and to assess the pH stability of the system, gels were swollen and freeze-dried for three cycles (Figure A1).

## Appendix B

In order to find the most suitable function to describe the release behavior of the hydrogels mathematically, the data were plotted according to common models (Table 1) in kinetic drug delivery studies (Table A1). As *k*_0_ values of Peppas–Sahlin’s model were close to zero (equivalent to Korsmeyer–Peppas’ model) or negative, they were not included in the comparison.

**Table A1:** Exponential values *n* of drug release mechanisms for different hydrogel geometries.

pH		HA_35_		HA_55_	
		10 kDa Dextran	40 kDa Dextran	10 kDa Dextran	40 kDa Dextran
5.0	Model	Second-order	Logarithmic	Logarithmic	Logarithmic
	Parameters	*k*_2_ = 0.036 ± 0.003	*a* = 14.0 ± 1.0	*a* = 24.4 ± 1.7	*a* = 16.8 ± 1.6
			*b* = −16.4 ± 1.7	*b* = 22.7 ± 2.6	*b* = −11.5 ± 3.7
	*R* ^2^	0.9340	0.9633	0.9708	0.9406
7.4	Model	Logarithmic	Second-order	Logarithmic	Logarithmic
	Parameters	*a* = 42.4 ± 2.9	*k*_2_ = 0.0057 ± 0.0003	*a* = 49.9 ± 2.1	*a* = 37.9 ± 1.8
		*b* = −32.8 ± 4.8		*b* = −40.6 ± 3.1	*b* = −37.9 ± 3.5
	*R* ^2^	0.9771	0.9923	0.9926	0.9874
10.0	Model	Logarithmic	Korsmeyer–Peppas	Korsmeyer–Peppas	Korsmeyer–Peppas
	Parameters	*a* = 46.0 ± 3.5	*k*_p_ = 0.031 ± 0.006	*k*_p_ = 0.086 ± 0.012	*k*_p_ = 0.042 ± 0.007
		*b* = −28.8 ± 6.9	*n* = 0.51 ± 0.04	*n* = 0.39 ± 0.03	*n* = 0.48 ± 0.03
	*R* ^2^	0.9669	0.9732	0.9744	0.9795

Conclusions about the type of transport were drawn from Korsmeyer–Peppas and Peppas–Sahlin plots (Table A2). Here, the exponent *n* of Korsmeyer–Peppas’ model and the Peppas–Sahlin rate constants *k*_0_ were thoroughly examined.

**Table A2:** Exponential values *n* of drug release mechanisms for different hydrogel geometries.

pH	Parameter	HA_35_		HA_55_	
		10 kDa Dextran	40 kDa Dextran	10 kDa Dextran	40 kDa Dextran
5.0	*n*	0.22 ± 0.04	0.56 ± 0.11	0.16 ± 0.01	0.33 ± 0.05
	*k*_0_	−0.0033 ± 0.0002	0.0002 ± 0.0005	−0.0051 ± 0.0007	−0.0014 ± 0.0004
	Mechanism	(hind.) Fickian	Fickian to anomal.	(hind.) Fickian	(hind.) Fickian
7.4	*n*	0.38 ± 0.03	0.57 ± 0.06	0.47 ± 0.03	053 ± 0.05
	*k*_0_	−0.0010 ± 0.0002	0.0002 ± 0.0003	−0.0004 ± 0.0003	0.0001 ± 0.0003
	Mechanism	(hind.) Fickian	Fickian to anomal.	Fickian to anomal.	Fickian to anomal.
10.0	*n*	0.38 ± 0.04	0.51 ± 0.04	0.39 ± 0.03	0.48 ± 0.03
	*k*_0_	−0.0011 ± 0.0003	0.0001 ± 0.0002	−0.0010 ± 0.0003	−0.0001 ± 0.0002
	Mechanism	(hind.) Fickian	Fickian to anomal.	(hind.) Fickian	Fickian to anomal.

## Appendix C

Concentrations of FITC-dextrans in buffered solutions were determined through fluorescence measurements. For each buffer, an individual standard curve (Figure A2) was prepared. For samples at pH 5.0 mixed with Tris buffer for analysis, the standard curve (Figure A2a) was prepared accordingly.
